# Effect of Different Adjuvants on the Longevity and Strength of Humoral and Cellular Immune Responses to the HCV Envelope Glycoproteins

**DOI:** 10.3390/vaccines7040204

**Published:** 2019-12-03

**Authors:** Bassel Akache, Lise Deschatelets, Blair A. Harrison, Renu Dudani, Felicity C. Stark, Yimei Jia, Amir Landi, John L. M. Law, Michael Logan, Darren Hockman, Juthika Kundu, D. Lorne Tyrrell, Lakshmi Krishnan, Michael Houghton, Michael J. McCluskie

**Affiliations:** 1 National Research Council Canada, Human Health Therapeutics, 1200 Montreal Rd, Ottawa, ON K1T 0H1, Canada; bassel.akache@nrc-cnrc.gc.ca (B.A.); lise.deschatelets@nrc-cnrc.gc.ca (L.D.); blair.harrison@nrc-cnrc.gc.ca (B.A.H.); renu.dudani@nrc-cnrc.gc.ca (R.D.); felicity.stark@nrc-cnrc.gc.ca (F.C.S.); yimei.jia@nrc-cnrc.gc.ca (Y.J.); lakshmi.krishnan@nrc-cnrc.gc.ca (L.K.); 2 Li Ka Shing Institute of Virology, Department of Medical Microbiology & Immunology, University of Alberta, 6-010 Katz Group-Rexall Centre for Health Research, Edmonton, AB T6G 2E1, Canada; landi@ualberta.ca (A.L.); llaw@ualberta.ca (J.L.M.L.); logan@ualberta.ca (M.L.); darren.hockman@ualberta.ca (D.H.); juthika@ualberta.ca (J.K.); lorne.tyrrell@ualberta.ca (D.L.T.); mhoughto@ualberta.ca (M.H.)

**Keywords:** hepatitis C, glycoprotein E1/E2, archaeosome, SLA, vaccine, adjuvant, glycolipid

## Abstract

Infection by Hepatitis C virus (HCV) can lead to liver cirrhosis/hepatocellular carcinoma and remains a major cause of serious disease morbidity and mortality worldwide. However, current treatment regimens remain inaccessible to most patients, particularly in developing countries, and, therefore, the development of a novel vaccine capable of protecting subjects from chronic infection by HCV could greatly reduce the rates of HCV infection, subsequent liver pathogenesis, and in some cases death. Herein, we evaluated two different semi-synthetic archaeosome formulations as an adjuvant to the E1/E2 HCV envelope protein in a murine model and compared antigen-specific humoral (levels of anti-E1/E2 IgG and HCV pseudoparticle neutralization) and cellular responses (numbers of antigen-specific cytokine-producing T cells) to those generated with adjuvant formulations composed of mimetics of commercial adjuvants including a squalene oil-in-water emulsion, aluminum hydroxide/monophosphoryl lipid A (MPLA) and liposome/MPLA/QS-21. In addition, we measured the longevity of these responses, tracking humoral, and cellular responses up to 6 months following vaccination. Overall, we show that the strength and longevity of anti-HCV responses can be influenced by adjuvant selection. In particular, a simple admixed sulfated S-lactosylarchaeol (SLA) archaeosome formulation generated strong levels of HCV neutralizing antibodies and polyfunctional antigen-specific CD4 T cells producing multiple cytokines such as IFN-γ, TNF-α, and IL-2. While liposome/MPLA/QS-21 as adjuvant generated superior cellular responses, the SLA E1/E2 admixed formulation was superior or equivalent to the other tested formulations in all immune parameters tested.

## 1. Introduction

Hepatitis C virus (HCV) is a highly pathogenic virus infecting over 70 million people globally [1]. Chronic infection can lead to liver cirrhosis and hepatocellular carcinoma, causing approximately ~400,000 deaths per year worldwide. As viral transmission occurs through exposure to contaminated blood, the recent surge in intravenous illicit drug use in the US has also led to a concomitant increase in the rates of HCV infection [2]. Antiviral drugs such as Vesovi^®^ (sofosbuvir, velpatasvir, and voxilaprevir combination regimen) have been recently approved by the FDA for the treatment of HCV infection and have demonstrated >95% cure rate in clinical trials [3]. However, low rates of disease diagnosis accompanied by the high cost of the treatment regimen could restrict access to the drug for a large segment of the affected population, particularly in developing countries.

Vaccines remain amongst the most efficacious and cost-effective approaches to reduce rates of infectious disease transmission. Multiple HCV vaccine candidates have been evaluated in non-human primates and/or human clinical trials [4]. MF59^®^-adjuvanted vaccines targeting glycoproteins E1 and E2 on the viral envelope surface have been shown to suppress viral infection in chimpanzees and generate neutralizing antibodies in human subjects [5,6]. Antigen-specific CD4 helper T cells were also detected in the vaccinated subjects. Importantly, the antibodies generated in the clinical trial were cross-neutralizing. Although the vaccine antigens were derived from a single HCV genotype (1a), antibodies in certain patients were capable of neutralizing infection by multiple clades of HCV in vitro [7]. Interestingly, the only HCV vaccine candidate shown to significantly reduce the incidence of chronic persistent infection in the chimpanzee model after prophylactic immunization and experimental challenge using either homologous or heterologous HCV 1a is a formulation consisting of E1/E2 antigen derived from a single 1a genotype combined with oil-in-water-based adjuvants [8,9].

While selecting the proper antigen is crucial for the development of an efficacious HCV vaccine, it is also important to include an appropriate adjuvant ideally capable of inducing robust, long-lasting, humoral, and cell-based immune responses. Archaeal lipid-based adjuvants have been previously shown to induce both antibody and cellular immune responses against multiple antigens, including ovalbumin and Hepatitis B surface antigen (HBsAg) in preclinical mouse models [10,11,12,13]. They have not been tested clinically yet. Traditional archaeosomal formulations consisted of liposomes formed from total polar lipids (TPL) derived from archaea such as the methanogen Methanobrevibacter smithii (MS). These archaeosomes were shown to effectively activate professional antigen-presenting cells [14,15,16] and generate robust cellular and humoral immune response to encapsulated antigen in both cancer and infectious disease models [11,12,17]. More recently, a novel simpler semi-synthetic archaeosome formulation composed of a sulfated disaccharide group covalently linked to the free sn-1 hydroxyl backbone of an archaeal core lipid (sulfated S-lactosylarchaeol, SLA), has been developed [18]. Archaeosomes formed with SLA alone or in combination with lactosylarchaeol (LA) retain a similar level of adjuvanticity to MS TPL archaeosomes, but consist of a simpler formulation with many advantages including consistency of production, reduced costs, and ease of synthesis [10,14,19]. A recent comparison of SLA to a wide panel of commercial adjuvants (e.g., aluminum salts, TLR agonists, and water/oil emulsions) when paired with the model antigens ovalbumin and HBsAg demonstrated that SLA archaeosome formulations had robust adjuvant activity that was superior to many of the other tested adjuvants [10]. Interestingly, a novel SLA archaeosome formulation, whereby the antigen is simply admixed with preformed SLA archaeosomes, has also been shown to stimulate equal or superior antigen-specific responses as formulations where the antigen is entrapped within the archaeosome [20]. As the efficiency of antigen entrapment within archaeosomes formulations is typically variable and relatively low (5–40%) [21,22], this new admixed formulation provides a convenient easy to mix format with no loss of antigen during the formulation process, thereby reducing costs and standardizing the amount of archaeal lipid in the final vaccine formulations.

Previous studies evaluated the immunogenicity of the HCV E1/E2-based vaccine with a panel of commercial and experimental adjuvants [13], including a squalene based oil-in-water emulsion used as a mimetic of MF59^®^ (the oil-in-water emulsion found in the marketed influenza vaccine Fluad™) and Alum/MPL, an aluminum salt combined with the TLR4 agonist Monophosphoryl lipid A (MPL) as a mimetic of AS04™ (the adjuvant combination contained in the commercial Hepatitis B vaccine Fendrix™) [23,24]. In addition, SLA/LA-based archaeosomes and cyclic di-AMP STING agonists were also evaluated in the same study [13]. These latter two adjuvant formulations induced superior E1/E2 immune responses, generating more robust antigen-specific T cell responses. Herein, we wanted to extend these studies to evaluate further our novel admixed SLA archaeosome formulation and also compare with a mimetic of AS01™ (an adjuvant system found in in the newly approved Shingles vaccine, Shingrix™ that combines two immunostimulatory molecules, MPL, and the saponin QS-21, with liposomes) [25]. In addition, the longevity of the E1/E2 humoral and cellular responses was evaluated to ensure that the tested adjuvants were capable of inducing long-lasting HCV-specific immune responses. Overall, it is shown that the admixed SLA formulation, along with the AS01™ and MF59^®^ mimetics, induce robust and long-lived immune responses detectable in mice up to 6 months following the last vaccination.

## 2. Materials and Methods:

### 2.1. Mice

6–8 week old female C57BL/6 x BALB/c F1 mice were obtained from Charles River Laboratories (Saint-Constant, Canada). Mice were maintained at the small animal facility of the National Research Council Canada (NRC) in accordance with the guidelines of the Canadian Council on Animal Care. All procedures performed on animals in this study were in accordance with regulations and guidelines reviewed and approved in animal use protocol 2016.08 by the NRC Human Health Therapeutics Animal Care Committee.

### 2.2. Vaccine Preparation

Vaccine antigen consisting of HCV H77 recombinant E1/E2 heterodimer was prepared as previously described [26]. 1 µg of E1/E2 was diluted in phosphate-buffered saline (PBS; Thermo Fisher Scientific, Waltham, MA, USA) and administered alone or in combination with various adjuvants in a final volume of 50 µL. E1/E2 encapsulated within SLA archaeosomes, SLA (Enc), or simply admixed with pre-formed empty archaeosomes, SLA (Adm) was prepared as previously described [20] and contained 0.115 and 1 mg of SLA per dose, respectively. AddaVax™ (Invivogen, San Diego, CA, USA), a mimetic of the squalene-oil-in-water emulsion adjuvant MF-59, was admixed 1:1 *v*/*v* with E1/E2. Aluminum hydroxide/monophosphoryl lipid A (alum/MPLA), a mimetic of the AS04™ adjuvant formulation was prepared as described previously [13] using alum (Alhydrogel^®^ “85”, aluminum hydroxide, 100 µg Al^3+^, Brenntag Biosector, Frederikssund, Denmark), and MPL (TLR4 agonist—monophosphoryl Lipid A from S. minnesota R595 VacciGrade, 10 µg, Invivogen), prepared as per manufacturer’s instructions and combined prior to the addition of E1/E2. Finally, a liposome/MPLA/QS-21 formulation was prepared as a mimetic for AS01B based on published methods [27]. In brief, E1/E2 was incorporated into liposomes composed of L-α-phosphatidylcholine derived from egg (Millipore Sigma, Oakville, ON, Canada) and cholesterol (Millipore Sigma). Non-entrapped E1/E2 was removed by centrifugation and liposomes washed in water. The E1/E2 concentration was determined by gel electrophoresis using densitometry, and the solution diluted to 40 µg/mL E1/E2. Finally, QS-21 (Desert King International, San Diego, CA, USA) and MPLA (Invivogen) were added to the E1/E2-containing liposomes at a final concentration of 100 µg/mL each, diluting the E1/E2 down to a final concentration of 20 µg/mL. As such, each vaccine dose contained 1 µg of E1/E2and 5 µg of each adjuvant (i.e., MPLA and QS-21). Adjuvant dose levels were based on data from previous studies.

### 2.3. Immunization of Mice and Sample Collection

Mice (n = 10/group) were immunized by intramuscular (i.m.) injection (50 µL) into the left tibialis anterior (T.A.) muscle on days 0, 21, and 35 with a total dose per injection of 1 µg HCV E1/E2 alone or formulated with the various adjuvant formulations. Negative control groups consisted of unimmunized naïve mice. Groups contained 2 cohorts of 5 animals with Cohort 1 euthanized on day 42 to evaluate cellular responses 7 days following final vaccination, and Cohort 2 euthanized on day 224 to evaluate the longevity of cellular responses approximately 6 months later. To recall the antigen-specific T cells, all animals in Cohort 2, regardless of group, were injected i.m. with 0.2 µg of antigen alone on day 220. Spleens were collected from euthanized animals for measurement of cellular immune responses by IFN-γ ELISpot and/or intracellular cytokine staining. Animals were bled via the submandibular vein on Days 20, 42, 121, 219 and 224, and recovered serum was used for quantification of antigen-specific IgG antibody levels.

### 2.4. Anti-E1/E2 ELISA

Anti-E1/E2 total IgG titers in mouse serum were quantified by ELISA. Briefly, 96–well high-binding ELISA plates (Thermo Fisher Scientific) were coated overnight at room temperature (RT) with 100 µL of 0.15 µg/mL E1/E2 protein (same as used for immunization) diluted in PBS. Plates were washed 5 times with PBS/0.05% Tween20 (PBS-T; Sigma-Aldrich, St. Louis, Missouri, USA), and then blocked for 1 h at 37 °C with 200 µL 10% fetal bovine serum (FBS; Thermo Fisher Scientific) in PBS. After the plates were washed 5 times with PBS-T, 3.162-fold serially diluted samples in PBS-T with 10% FBS was added in 100 µL volumes and incubated for 1 h at 37 °C. After 5 washes with PBS-T (Sigma-Aldrich), 100 µL of goat anti-mouse IgG -HRP (1:4000, Southern Biotech, Birmingham, AL USA) was added for 1 h at 37 °C. After 5 washes with PBS-T, 100 µL/well of the substrate o-phenylenediamine dihydrochloride (OPD, Sigma-Aldrich) diluted in 0.05 M citrate buffer (pH 5.0) was added. Plates were developed for 30 min at RT in the dark. The reaction was stopped with 50 µL/well of 4N H_2_SO_4_. Bound IgG Abs were detected spectrophotometrically at 450 nm. Titers for IgG in serum were defined as the dilution that resulted in an absorbance value (OD 450) of 0.2 and was calculated using XLfit software (ID Business Solutions, Guildford, UK). No detectable titers were measured in serum samples from naïve control animals.

### 2.5. HCV Pseudoparticle Neutralization Assay

The neutralization assay was performed as described previously [13]. Briefly, the ability of HCV pseudoparticles (HCVpp; retroviral vectors encoding luciferase and expressing HCV E1/E2 proteins on the capsid surface) to transduce Huh7.5 liver cell lines following incubation with sera from immunized mice were measured. Serum was heat-inactivated at 56 °C for 30 min and diluted 1:250 in Dulbecco’s modified eagle medium (DMEM; Thermo Fisher Scientific) containing 3% FBS (Thermo Fisher Scientific), 1% penicillin/streptomycin (P/S; Thermo Fisher Scientific), 20 mM HEPES (Thermo Fisher Scientific), and 4 µg/mL polybrene (Merck Millipore, Burlington, MA, USA). An equal volume of HCVpp was added and samples incubated for 1 h at 37 °C with 5% CO_2_. Huh7.5 cells cultured on 96-well plates were spinoculated with the HCVpp:serum mixtures by centrifugation for 1 h at RT at 1200 rpm. Plates were incubated at 37 °C with 5% CO_2_. After 5 h, the media was removed, and 100 µL of DMEM containing 10% FBS, 1% P/S, 1% glutamine, and 1% non-essential amino acids (Thermo Fisher Scientific) was added. After ~44 h, the cells were washed with PBS and lysed with 50 µL of lysis buffer (Promega Corporation, Madison, WI, USA) at room temperature for 5 min. 50 µL of luciferin (Promega Corporation) was then added, and luminescence of the solution was measured 15 min later using a luminescence plate reader (Tecan Group Limited, Männedorf, Switzerland) in 96-well white Falcon^®^ plates (Corning, Tewksbury, MA, USA). Percent neutralization was calculated as follows: % neutralization = 100 − (100 × (luminescence in the presence of mouse serum)/(luminescence in the absence of mouse serum)). For analysis purposes, samples with calculated values ≤1 were assigned a value of 1.

### 2.6. ELISpot

The levels of E1/E2 specific T cells were quantified by ELISpot using a mouse IFN-γ kit (Mabtech Inc., Cincinnati, OH, USA). Spleens were mechanically minced with the frosted ends of two glass slides and splenocytes were isolated in Roswell Park Memorial Institute (RPMI) media (Thermo Fisher Scientific) containing 10% FBS (Thermo Fisher Scientific), 1% penicillin/streptomycin (Thermo Fisher Scientific), 1% glutamine (Thermo Fisher Scientific), and 55 µM 2-Mercaptoethanol (Thermo Fisher Scientific). Cells were passed through a 70 µm cell strainer and cell yields determined on a Cellometer (Nexcelom, Lawrence, MA). 4 × 10^5^ cells were stimulated in duplicate with an E1/E2 peptide library (GL Biochem Ltd., Shanghai, China) consisting of 55 20mer peptides overlapping by 10 amino acids at a concentration of 5 µg/mL. The final volume per well was 0.2 mL. Cells were also incubated without any stimulants to measure background responses. Plates were incubated for ~20 h at 37 °C with 5% CO_2_, at which point the plates were washed and developed according to the manufacturer’s instructions. AEC substrate (Becton Dickenson, Franklin Lakes, NJ, USA) was used to visualize the spots. Spots were counted using an automated ELISpot plate reader (Zellnet consulting, Fort Lee, NJ, USA).

### 2.7. Intracellular Cytokine Staining

The phenotype (CD4 vs. CD8) and polyfunctionality (expression of IFN-γ, TNF-α, and/or IL-2) of E1/E2 specific T cells were determined by intracellular cytokine staining of splenocytes. Cells (2 × 10^6^ per sample) were stimulated with the E1/E2 peptide pool as described above in the presence of Golgiplug™ (Becton Dickenson) for ~20 h at 37 °C with 5% CO_2_. Cells were also incubated without any peptides to measure background responses. Following incubation, splenocytes were washed with PBS (Thermo Fisher Scientific) and stained with the fixable blue dead cell stain (Thermo Fisher Scientific). Cells were then stained with an antibody cocktail to identify immune cell types through binding of cell surface markers: Anti-CD14-BV510 (Becton Dickenson), anti-CD16-BV510 (Becton Dickenson), α-CD19-BV510 (Becton Dickenson), anti-CD4-APC-Cy7 (Becton Dickenson), and anti-CD8-PerCp-Cy5.5 (Becton Dickenson) diluted in staining buffer (PBS+ 2% FBS; Thermo Fisher Scientific). Cells were then washed in staining buffer and permeabilized for intracellular staining using the BD Cytofix/Cytoperm™ kit (Becton Dickenson) according to the manufacturer’s instructions. Samples were then stained with an antibody cocktail to anti-CD3-AF700 (eBioscience, San Diego, CA, USA), anti-CD69-PE-CF594 (Becton Dickenson), anti-IFN-γ-AF488 (Becton Dickenson), anti-TNF-α-BV421 (Becton Dickenson), anti-IL-2-APC (Becton Dickenson), and Granzyme B-PE-Cy7 (eBioscience) diluted in permeabilization wash buffer (Becton Dickenson). All samples were washed and resuspended in staining buffer for acquisition with a BD Fortessa flow cytometer (Becton Dickenson). Cell populations were characterized as follows: Non-T cells and dead cells were excluded based on staining for BV510 and the fixable dye, respectively. Activated CD3+CD4+ or CD3+CD8+ T cells were identified through positive staining of the CD69 activation marker prior to classifying them as IFN- γ, TNF-α, and/or IL-2 positive cells. Expression of the cytolytic marker Granzyme B was also evaluated on cytokine-producing CD8 T cells. The general gating strategy used to identify cytokine positive T cells is depicted in Figure S1.

### 2.8. Statistical Analysis

Data were analyzed using GraphPad Prism^®^ version 8 (GraphPad Software, San Diego, CA). Statistical significance of the difference between groups was calculated by one-way analysis of variance (ANOVA) followed by post-hoc analysis using Dunnett’s (comparison with control unadjuvanted group) multiple comparison test. Antibody titers and cytokine levels were log-transformed prior to statistical analysis. For all analyses, differences were considered to be not significant with *p* > 0.05. Significance was indicated in the graphs as follows: * *p* < 0.05, ** *p* < 0.01, *** *p* < 0.001 and **** *p* < 0.0001. Correlation between data sets was also determined using GraphPad Prism by calculating the Pearson correlation coefficient. Unless otherwise indicated in the figure legends, grouped data were presented as the mean + standard error of the mean (SEM).

## 3. Results

### 3.1. Humoral Response to E1/E2-Adjuvanted Vaccine Formulations in Mice

**Figure 1:**
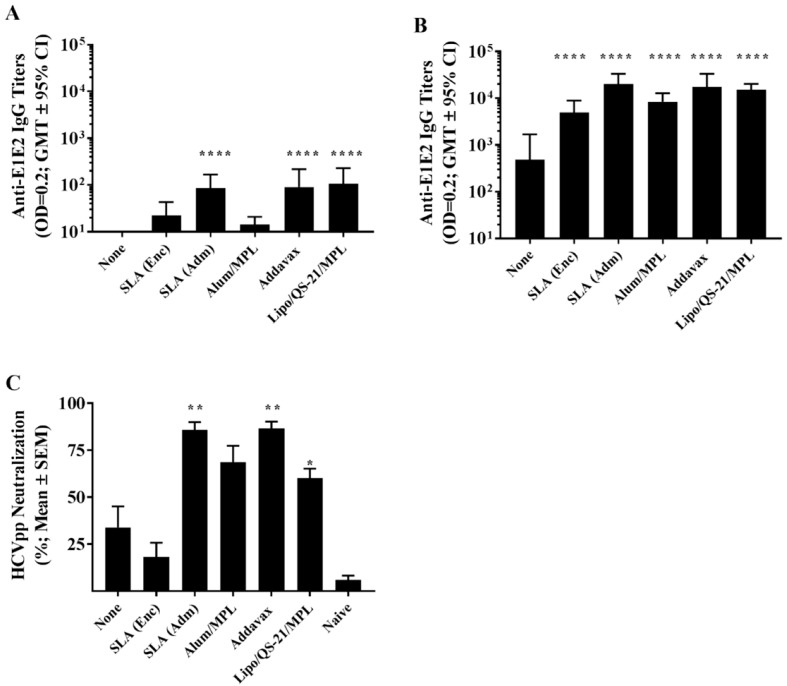
Anti-E1/E2 IgG titers and Hepatitis C virus (HCV) neutralization activity in the serum of immunized mice. C57Bl/6 x Balb/c F1 mice (n = 10/group) were immunized intramuscular (i.m.) with HCV E1/E2 protein (1 µg) with or without adjuvant on days 0, 21, and 35. Serum was obtained from all mice on days 20 (**A**) and 42 (**B**) with serum analyzed for anti-E1/E2 IgG antibody by ELISA. Grouped data is presented as GMT + 95% CI. Serum from Day 42 was also tested for its ability to neutralize HCVpp entry into Huh7.5 liver cells in vitro (**C**). Meanings of the asterisks show in Section 2.8.

Mice were immunized on days 0, 21, and 35 with E1/E2 antigen alone or in combination with different adjuvants including Addavax (an MF59^®^ mimetic), Alum/MPL (AS04™ mimetic), Lipo/QS-21/MPL (AS01™ mimetic), and E1E2 either encapsulated within SLA archaeosomes, SLA (Enc), or simply admixed with pre-formed empty archaeosomes, SLA (Adm). Anti-E1/E2 titers were assessed on day 20 (20 days post single vaccination) and on day 42 (7 days post third vaccination). Following a single vaccine dose, no measurable antibody titers were detected with the unadjuvanted formulation, while all the adjuvanted formulations had geometric mean titers (GMT) >10 (the lower limit of detection of the assay; Figure 1A). The titers in mice immunized with E1/E2 adjuvanted with SLA (Adm), Addavax, and Lipo/QS-21/MPL were higher than those obtained in mice immunized with antigen alone (*p* < 0.0001) with GMT (lower and upper 95% confidence interval (CI)) of 85.5 (43.5 and 168.1), 90.9 (38 and 217.1), and 106.4 (49.6 and 228.5), respectively.

On day 42, following an additional two vaccine doses, titers in all groups increased (Figure 1B). The largest fold-increase was seen with Alum/MPL, with a ~580-fold increase in GMT from day 20 to 42. Meanwhile, repeated vaccination with the other adjuvanted formulations yielded an ~140 to 230-fold increase in GMT over the same timeframe. All adjuvanted formulations induced E1/E2-specific IgG titers greater than those observed with antigen alone on day 42 (*p* < 0.0001). Again, the highest titers were observed in mice immunized with E1/E2 adjuvanted with SLA (Adm), Addavax, and Lipo/QS-21/MPL with GMT (lower and upper 95% CI) of 19,836 (11,794 and 33,360), 17,390 (9,118 and 33,165), and 15,054 (11,194 and 20,244), respectively.

Importantly, these antibodies were capable of neutralizing HCV pseudoparticles and preventing their entry into a human-derived liver cell line in vitro. In a HCV neutralization assay, expression of a reporter gene was inhibited significantly by sera from mice immunized using SLA (Adm) (*p* < 0.01), Addavax (*p* < 0.01) and Lipo/QS-21/MPL (*p* < 0.05) as adjuvants (Figure 1C) with a mean % reduction (SEM) of 85.8% (4.1), 86.6% (3.6) and 60.1% (5.1), respectively. While sera from E1/E2-Alum/MPL immunized mice did result in 68.6% (8.8) neutralization, this did not reach a level of statistical significance. The level of neutralization with sera from unadjuvanted and SLA (Enc) -E1/E2 formulations were similarly low at 34% (11.1) and 18.2% (7.6), respectively.

### 3.2. Cellular Response to E1/E2-Adjuvanted Vaccine Formulations in Mice

**Figure 2:**
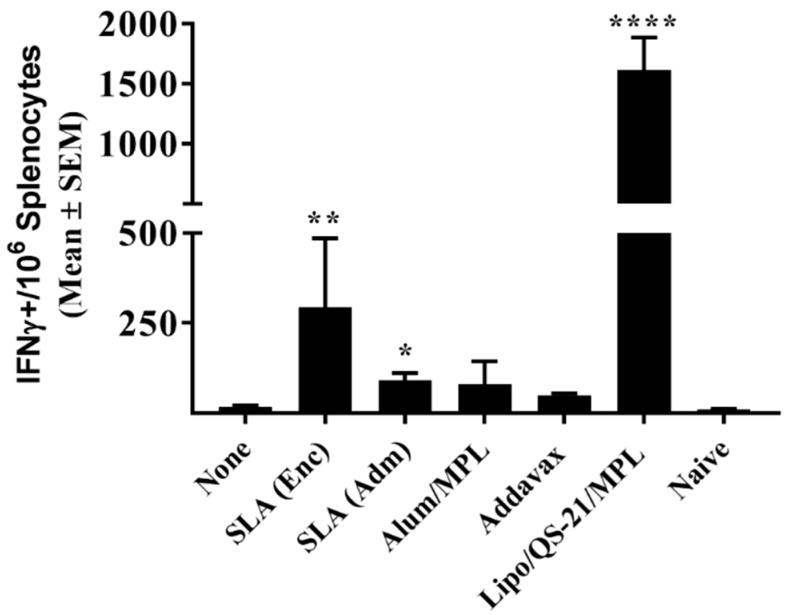
E1/E2-specific T cells as determined by IFN- γ ELISpot with splenocytes of immunized mice. C57Bl/6 x Balb/c F1 mice were immunized i.m. with HCV E1/E2 protein (1 µg) with or without adjuvant on days 0, 21, and 35. Splenocytes were harvested on day 42 (n = 5/group) and analyzed by IFN-γ ELISpot when stimulated by media alone or an E1/E2 peptide pool. Values obtained with media alone were subtracted from those measured in the presence of the peptide pool. Meanings of the asterisks show in Section 2.8.

E1/E2-specific T cell responses were assessed through IFN-γ ELISpot and intracellular cytokine staining (ICCS). Seven days following the third vaccination, splenocytes from mice immunized with Lipo/QS-21/MPL had significantly higher levels of IFN-γ+ T cells induced by a E1/E2 peptide library than those obtained in the unadjuvanted group with a group mean (SEM) of 1616 (269) vs. 17.5 (3.9) IFN-γ positive spot-forming cells (SFC)/10^6^ splenocytes, (*p* < 0.0001; Figure 2). SLA (Enc) and SLA (Adm)-adjuvanted formulations also induced significantly higher numbers of IFN-γ positive cells/10^6^ splenocytes than antigen alone with a mean (SEM; *p*-value) of 294 (191; *p* < 0.01) and 91 (21; *p* < 0.05), respectively.

**Figure 3:**
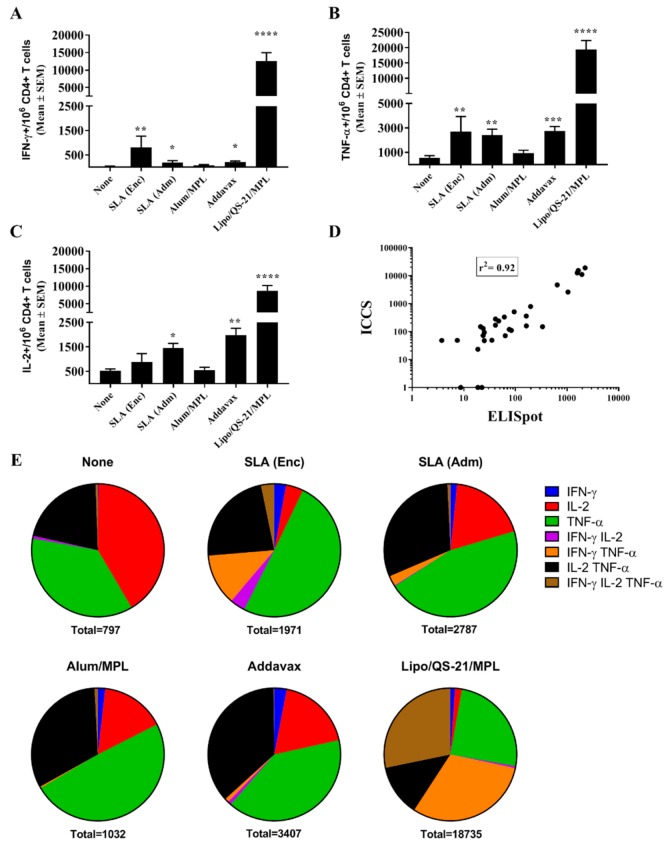
Polyfunctionality and phenotype of E1/E2-specific T cells as determined by intracellular cytokine staining of splenocytes of immunized mice. C57Bl/6 x Balb/c F1 mice were immunized i.m. with HCV E1/E2 protein (1 µg) with or without adjuvant on days 0, 21, and 35. Splenocytes were harvested on day 42 (n = 5/group) and analyzed by intracellular cytokine staining (ICCS) when stimulated by an E1/E2 peptide pool or media alone. The number of E1/E2-specific IFN-γ+ (**A**), TNF-α+ (**B**), and IL-2+ (**C**) per million CD4 T cells were determined. Values obtained with media alone were subtracted from those measured in the presence of the peptide pool. Correlation of IFN-γ responses as determined by ELISpot and ICCS was also performed (**D**). The frequency of cells expressing IFN-γ, TNF-α, and IL-2 alone or in combination is displayed (geomean per group) with the total number of cytokine-positive cells per million CD4+ T cells indicated below the pie chart (**E**). Meanings of the asterisks show in Section 2.8.

When the same cells were analyzed by ICCS, it was confirmed that the Ag-specific T cells were of the CD4 phenotype, with no increased production of IFN-γ, TNF-α, or IL-2 seen in CD8 T cells stimulated with the E1/E2 peptide library in any groups. Within the CD4 T cell population, IFN-γ+ cells were observed across the various groups in a pattern similar to that seen with the ELISpot assay above (Figure 3A). Lipo/QS-21/MPL, SLA (Enc) and SLA (Adm) had significantly higher levels of IFN-γ+ CD4 T cells induced by a E1/E2 peptide library with mean (SEM; *p*-value) of 12,553 (269; *p* < 0.0001), 810 (463; *p* < 0.01) and 190 (82; *p* < 0.05), respectively, vs. 34 (14.2) in the unadjuvanted group. In addition, Addavax-adjuvanted E1/E2 induced significantly higher levels of IFN-γ positive cells/10^6^ CD4 T cells with 203 (51; *p* < 0.05). A strong correlation was observed between the levels of IFN-γ obtained by ELISpot and ICCS for individual mice (r^2^ = 0.92; Figure 3D). A significant increase in Ag-specific TNF-α and IL-2 production in response to E1/E2 stimulation was observed in CD4 T cells of mice immunized with SLA (Enc), SLA (Adm), Addavax, and Lipo/QS-21/MPL-adjuvanted formulations, with one exception (Figure 3B,C). While levels of IL-2+ CD4+ T cells were slightly higher in the splenocytes of mice immunized with the SLA (Enc)-E1/E2 formulation when compared to the unadjuvanted control (mean (SEM) of 884 (346) vs. 528 (74)) they did not reach a level of statistical significance. A large proportion of the antigen-specific CD4 cells were positive for multiple cytokines (i.e., 22–72%; Figure 3E). The percentage of cytokine-positive CD4 T cells expressing more than one cytokine in mice immunized with SLA (Enc), SLA (Adm), Addavax, and Lipo/QS-21/MPL -adjuvanted formulations was 42%, 34%, 37%, and 72%, respectively.

### 3.3. Longevity of E1/E2-Specific Humoral and Cellular Immune Responses in Mice

**Figure 4:**
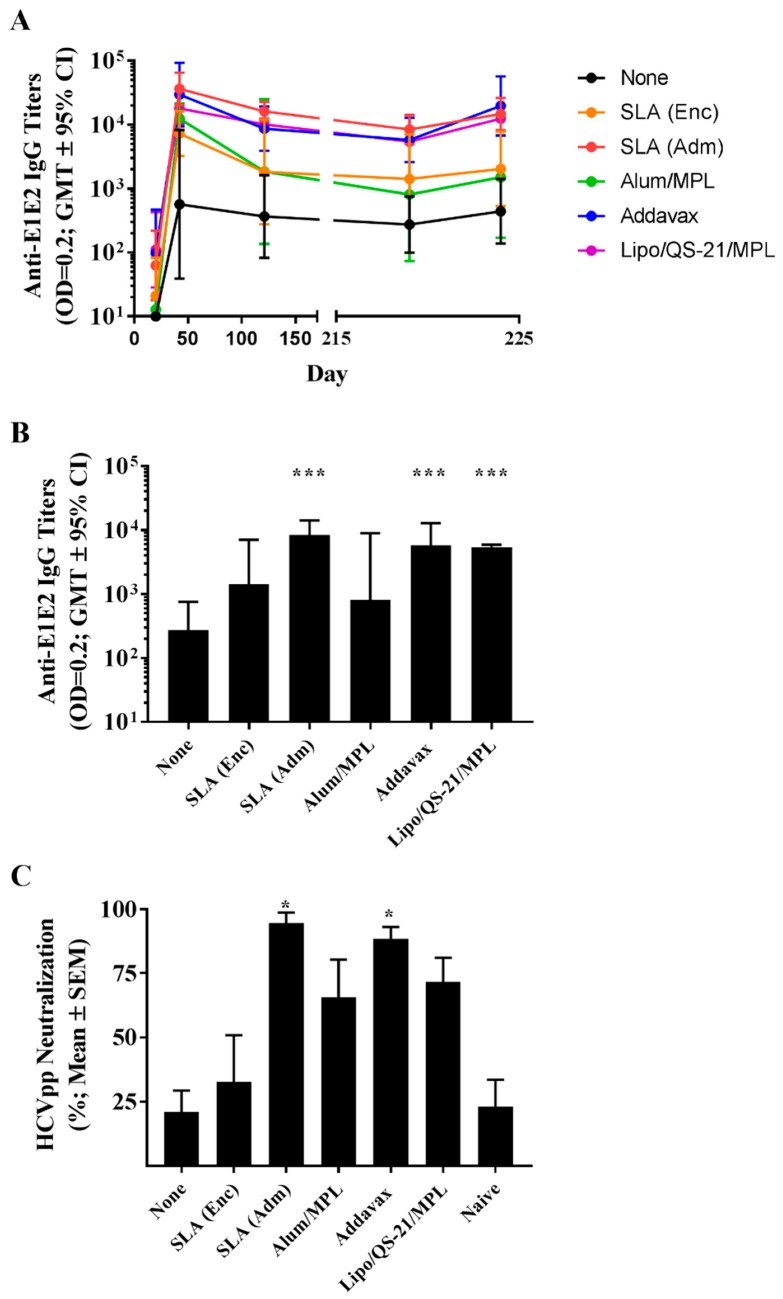
Longevity of anti-E1/E2 IgG titers and HCV neutralization activity in serum of immunized mice. C57Bl/6 x Balb/c F1 mice were immunized i.m. with HCV E1/E2 protein (1 µg) with or without adjuvant on days 0, 21, and 35. Sera were obtained from mice (n = 5/group) at multiple timepoints (i.e., days 20, 42, 121, 219, and 224) to track anti-E1/E2 antibody titers over time. On day 220, mice were injected i.m. with 0.2 µg of E1/E2 without adjuvant to look at the antigen-specific recall response. Anti-E1/E2 IgG titers over the course of the study (**A**) and specifically on Day 219 (**B**) were determined by ELISA. Grouped data is presented as GMT + 95% CI. Serum from Day 219 was also tested for its ability to neutralize HCVpp entry into Huh7.5 liver cells in vitro (**C**). Meanings of the asterisks show in Section 2.8.

The longevity of immune responses generated by E1/E2-based vaccines was also assessed in mice to confirm the ability of the different adjuvants to generate long-lasting antigen-specific humoral and cellular immune responses. Mice were maintained for ~6 months post-last vaccination, and sera were collected at multiple timepoints (i.e., days 121, 219, and 224) to track anti-E1/E2 antibody titers over time. On day 220, mice received a low 0.2 µg dose of E1/E2 to assist in the evaluation of the antigen-specific recall response in the mice. The E1/E2-specific IgG titers did decrease over time with a 2 to 15-fold decrease in GMT from days 42 to 219 seen across the various groups (Figure 4A). The largest fold decrease was seen in animals immunized with the Alum/MPL-adjuvanted formulation (i.e., 15-fold), although it did not reach a level of statistical significance (*p* = 0.1316). Over the same timepoints, statistically significant decreases were seen with animals immunized with E1/E2 adjuvanted with either SLA (Adm) (4-fold, *p* < 0.05) and Lipo/QS-21/MPL (3-fold, *p* < 0.05). Interestingly, the administration of the low dose E1/E2 antigen on day 220 led to a significant rise in antibody titers in animals previously vaccinated with E1/E2 adjuvanted with SLA (Adm), Addavax or Lipo/QS-21/MPL with 2-fold (*p* < 0.01), 3-fold (*p* < 0.05) and 2-fold (*p* < 0.0001) increases in antibody GMT seen between days 219 and 224, respectively. Animals in the other study groups did not have statistically significant different antibody titers between these timepoints.

Importantly, antibodies in the serum of mice 6 months following last vaccination (i.e., from day 219) were still significantly higher in mice receiving E1/E2 adjuvanted with SLA (Adm), Addavax, or Lipo/QS-21/MPL vs. E1/E2 alone (Figure 4B; *p* < 0.001). In addition, serum from mice immunized with the SLA (Adm) and Addavax -adjuvanted formulations were capable of significantly neutralizing HCV pseudoparticles in vitro, with a mean % reduction (SEM) of 94.6% (4.1) and 88.4% (4.7), respectively, compared to 21% (8.4) with the unadjuvanted formulation (Figure 4C; *p* < 0.05).

**Figure 5:**
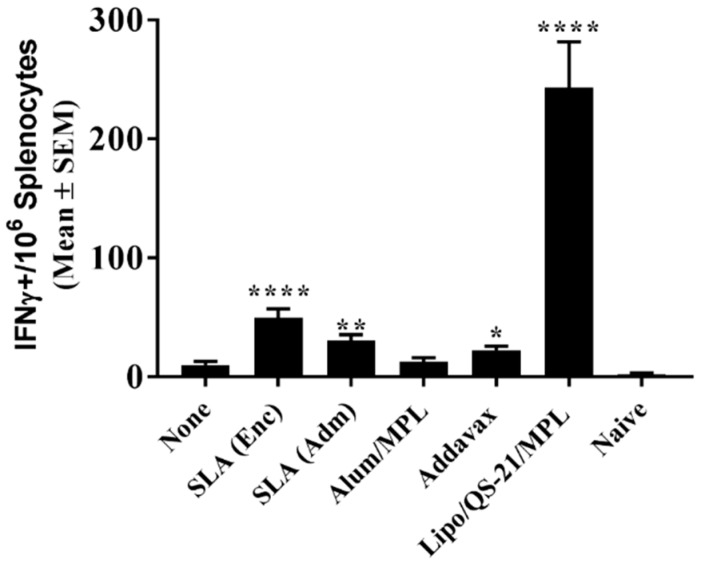
Longevity of E1/E2-specific T cell responses in splenocytes of immunized mice. C57Bl/6 x Balb/c F1 mice were immunized i.m. with HCV E1/E2 protein (1 µg) with or without adjuvant on days 0, 21, and 35. On day 220, mice were injected i.m. with 0.2 µg of E1/E2 without adjuvant. Splenocytes were harvested on Day 224 (n = 5/group) and analyzed by IFN-γ ELISpot when stimulated by media alone or an E1/E2 peptide pool. Values obtained with media alone were subtracted from those measured in the presence of the peptide pool. Meanings of the asterisks show in Section 2.8.

To confirm the longevity of the E1/E2-specific T cell responses, splenocytes collected on day 224 were stimulated with E1/E2-derived peptide pool and IFN-γ production measured by ELISpot. As on day 42, mice immunized with Lipo/QS-21/MPL had the highest levels of IFN-γ+ SFC/10^6^ splenocytes with group mean (SEM) of 242.8 (38.8) vs. 9.8 (3.1) obtained in the unadjuvanted group, (*p* < 0.0001; Figure 5). SLA (Enc), SLA (Adm), and Addavax-adjuvanted formulations also induced significantly higher numbers of IFN-γ positive cells/10^6^ splenocytes than immunization with antigen alone with group mean (SEM; *p*-value) of 49.5 (7.7; *p* < 0.0001), 30.3 (5.1; *p* < 0.01) and 22.3 (3.7; *p* < 0.05), respectively.

## 4. Discussion

The HCV E1/E2 heterodimer plays an essential role in virus entry, with E2 binding directly to the receptor CD81 on the surface of the host cell. While HCV-neutralizing antibodies have been shown to bind epitopes on E1 or E2, the majority of HCV-neutralizing antibodies in humans target E2, interrupting its interaction with its cell surface receptor [28]. E1/E2-based vaccines have been shown in a preclinical setting to induce robust immune responses with generated antibodies capable of neutralizing HCV in vitro and preventing infection in non-human primate models in vivo [5]. E1/E2 antigen derived from a single 1a genotype combined with oil-in-water-based adjuvants remains the only HCV vaccine candidate shown to significantly reduce the incidence of chronic persistent infection in the chimpanzee model after prophylactic immunization and experimental challenge using either homologous or heterologous 1a virus [8,9]. As with other subunit vaccines, a clinically efficacious E1/E2 vaccine formulation would likely require inclusion of an adjuvant capable of inducing strong and long-lived antigen-specific immune responses. In Phase I studies, a MF59^®^-adjuvanted E1/E2 vaccine formulation was well tolerated and induced antigen-specific lymphoproliferative and cross-neutralizing antibody responses in healthy human subjects [6,7,29]. The ability of this vaccine formulation to protect against chronic HCV infection has yet to be determined. Likewise, it is unknown whether superior immune responses would be obtained with other commercially approved adjuvants such as AS01™ and AS04™ if incorporated in an E1/E2-based vaccine. SLA-based adjuvants enhance the generation of antigen-specific immune responses through increased local cytokine production, immune cell trafficking, and antigen uptake at the injection site, leading to increased protection in murine models of infectious disease (e.g., influenza virus) and cancer [10,14,19,20,30]. In previous studies, an encapsulated SLA-based formulation was able to enhance E1/E2-specific cellular responses but did not significantly enhance antibody-mediated HCVpp neutralization [13]. Therefore, in this study we compared the ability of both the SLA (Enc) and SLA (Adm) formulations to enhance immune responses to E1/E2 with mimetics of three adjuvants currently used in approved vaccines, namely the squalene-based oil-in-water emulsion Addavax (a mimetic of MF59^®^), the TLR4 agonist MPL combined with aluminum salts (a mimetic of AS04™), and a liposome formulation containing MPL/QS-21 (a mimetic of AS01™). These adjuvants were selected as they are mimetics of adjuvants approved for human use and cover a range of different mechanisms of action. In addition, we sought to compare the longevity of the immune responses generated by these formulations, which has not been previously assessed for experimental vaccines containing SLA-based archaeosomes. Previously, the levels of antigen-specific antibodies and cellular responses were assessed within a few weeks of final immunization [10]. Long-lived immune responses are critical in the context of prophylactic vaccines where exposure to an infectious agent may occur years post-vaccination.

Multiple novel adjuvants have been approved for clinical use in the past 20 years. MF59^®^ has been licensed for use in Europe since 1997 and in the United States since 2015 as part of the influenza vaccine, Fluad^®^ [23]. The AS04™ adjuvant system, containing an endotoxin-derived TLR4 agonist, MPL, along with aluminum salt, is currently formulated in marketed vaccines such as Fendrix^®^ and Cervarix^®^ approved for the prevention of infections by pneumococcal bacteria and human papillomavirus, respectively [24]. Most recently, AS01™, containing MPL along with the strongly immunostimulatory saponin QS-21 isolated from the bark of the Quillaja saponaria tree, was approved as part of the varicella-zoster vaccine, Shingrix [31]. While these vaccine adjuvants are approved, they may not be suitable for all indications, as different types/magnitudes of immune response may be necessary for a specific vaccine. In addition, due to their proprietary nature, access to these adjuvants is largely restricted to the pharmaceutical companies that developed them. Our results demonstrate that SLA archaeosome formulations, in particular, the SLA (Adm), along with the mimetics of MF59^®^ and AS01™ were capable of inducing long-lived humoral and cellular E1/E2-specific immune responses. The humoral response (E1/E2-specific IgG titers and HCVpp neutralization) induced by formulations containing these three adjuvants was largely similar in most of the assessed readouts. As previously reported by Landi et al. [13], when compared to the antigen alone, an archaeosome formulation with an encapsulated antigen induced significantly higher titers of antigen-specific IgG antibodies, but levels of HCVpp neutralization were not significantly different between the two groups. Administration of a low antigen dose 6 months following last vaccination induced a significant and rapid increase in antibody titers in the groups immunized with vaccine formulations adjuvanted with SLA (Adm), Addavax or Lipo/QS-21/MPL, indicative of a functional memory response. As the levels of memory cells were not directly measured in this study, future studies could address this by looking at the impact of various adjuvants on memory cell formation. Although we did not evaluate the ability of the antigen-specific antibodies generated in this study to cross-neutralize HCVpp coated with the E1/E2 proteins from other HCV genotypes, human subjects immunized with the same E1/E2 antigens did generate antibodies capable of neutralizing multiple strains of HCV [7].

While antibodies would be the main mediators of HCV viral particle neutralization, a robust antigen-specific CD4 T cell response could contribute to the quality and longevity of the humoral response. In addition, cytotoxic CD4 and CD8 T cells targeting E1/E2 would facilitate the clearance of virally-infected cells in infected individuals. Both SLA formulations, along with Lipo/QS-21/MPL were able to significantly enhance the levels of antigen-specific T cells when compared to antigen alone as determined by IFN-γ ELISpot. When evaluated by ICCS, it appears that none of the vaccine formulations were able to induce antigen-specific CD8 T cells as the cytokine secretion in response to the E1/E2 peptide pool was restricted to T cells of the CD4 phenotype. This was not unexpected, as previous reports have shown that T cell responses to E1/E2 recombinant antigen, when combined with various adjuvants, were mainly CD4-specific, but that expression of the E1/E2 from a viral-based DNA vector could induce antigen-specific CD8 T cells [13,32]. SLA archaeosomes have been shown to be strong and consistent inducers of CD8 T cell responses to model antigens such as OVA and HBsAg. The lack of CD8 responses in our model may be due to the nature of the antigen platform and/or the lack of processed E1/E2 epitopes with high binding affinity to the MHC molecules expressed in the mouse model used. In contrast, strong CD4 T cell responses were seen with many of the adjuvanted vaccine formulations. These responses were polyfunctional with the antigen-specific expression of IFN-γ, TNF-α, and IL-2 seen in the CD4 T cells of vaccinated mice. Alum/MPL was the weakest inducer of T cell responses with no significant differences seen in any of the cellular immune readouts when compared to antigen alone. The AS01™ mimetic, Lipo/QS-21/MPL, was the strongest inducer of E1/E2-specific CD4 T cells in our system. This agrees with the known profile of AS01™, which has been shown to robustly induce CD4 T cells to malaria and varicella-zoster antigens in preclinical and clinical studies [31]. The SLA (Enc)-adjuvanted formulation did induce strong CD4-mediated expression of IFN-γ and TNF-α, as previously reported [13]. Meanwhile, the SLA (Adm) formulation induced a significant increase in antigen-specific IL-2 production in addition to IFN-γ and TNF-α. Increased polyfunctionality of T cells is associated with more efficacious immune responses to certain pathogens [33]. As such, the use of SLA (Adm) instead of SLA (Enc) may offer a further advantage in addition to the superior humoral responses discussed above. The observed differences in the immune response between the two formulations may be due to the SLA lipid dose and/or antigen context (i.e., free protein vs. incorporated within the liposome). It would be of interest in future studies to evaluate these factors more closely to determine if they are contributing to the differences in activity of these two archaeosome formulations. In addition, mice immunized with the Lipo/QS-21/MPL-adjuvanted formulation did induce a large number of antigen-specific CD4 T cells shortly after immunization, but these were largely short-lived (1616 vs. 243 SFC/10^6^ splenocytes at 1 week and 6 months post last vaccination, respectively). A decrease over the same two timepoints was also seen with mice receiving the SLA (Adm)-adjuvanted formulation, but the decrease was ~3-fold (from 91 to 30 SFC/10^6^ splenocytes). We are currently evaluating combinations of other immunostimulatory molecules with differing mechanisms of action (such as TLR agonists and/or saponins) with the SLA (Adm) formulation as this approach has been shown to play a role in the immunogenicity of AS01™ [31]. As cytotoxic CD4 T cells have been shown to target multiple viral pathogens [34], and the tested adjuvanted vaccine formulations (especially Lipo/QS-21/MPL) induced E1/E2-specific CD4 T cells, it is possible that these vaccines could induce both cytotoxic and humoral immune responses targeting HCV despite the lack of a detectable E1/E2-specific CD8 responses in our system. It would be of interest in future studies to determine if the SLA formulation could be adapted to adjuvant responses generated with nucleic acid-based vaccine vectors to increase the number of antigen-specific CD8 T cells to HCV E1/E2. In addition, future studies could compare responses using an SLA-adjuvanted protein vaccine with those induced by an E1/E2 expressing viral-based DNA vaccine.

In summary, we have previously demonstrated that SLA archaeosomes are capable of inducing strong humoral and cellular immune responses against multiple antigens [10,19,20]. We have also shown that they are well-tolerated with a favorable safety profile when administered intramuscularly in vivo and can stimulate strong local cytokine secretion, immune cell recruitment, and antigen uptake at the vaccination site [14]. Herein, we have shown that while the E1/E2 antigen was immunogenic when administered alone, the magnitude and longevity of the antigen-specific responses were significantly enhanced by the inclusion of certain adjuvants. SLA glycolipids, particularly the simple admixed SLA formulation, can induce potent and long-lived anti-HCV E1/E2 responses in mice. In addition, these responses were equivalent or better to those obtained with various adjuvants (except for cellular responses vs. Lipo/QS-21/MPL). In addition, the overall cost of preparing the SLA admixed formulation is much reduced when compared to the encapsulated formulation as there is no antigen loss vs. 60–95% antigen loss when preparing the antigen-encapsulated SLA archaeosomes. Due to the limited availability of commercial adjuvants and its strong immunostimulatory profile, further development and characterization of the robust SLA adjuvant system with HCV E1/E2 is warranted.

## Supplementary Materials

The following are available online at https://www.mdpi.com/2076-393X/7/4/204/s1, Figure S1: Gating strategy for intracellular cytokine staining data.
