# Supplementary material for: Effect of Different Adjuvants on the Longevity and Strength of Humoral and Cellular Immune Responses to the HCV Envelope Glycoproteins
Source: Vaccines (Basel). 2019 Dec 3;7(4):204. doi: 10.3390/vaccines7040204 (PMC6963754; doi:10.3390/vaccines7040204)
Supplement: Supplementary file 1 [file vaccines-07-00204-s001.pdf]

vaccines

MDPI

Article

Effect of Different Adjuvants on the Longevity and Strength of Humoral and Cellular Immune Responses to the HCV Envelope Glycoproteins

Bassel Akache [1], Lise Deschatelets [1], Blair A. Harrison [1], Renu Dudani [1], Felicity C. Stark [1], Yimei Jia [1], Amir Landi [2], John L. M. Law [2], Michael Logan [2], Darren Hockman [2], Juthika Kundu [2], D. Lorne Tyrrell [2], Lakshmi Krishnan [1], Michael Houghton [2] and Michael J. McCluskie [1,*]

[1] National Research Council Canada, Human Health Therapeutics, 1200 Montreal Rd, Ottawa, ON K1T 0H1, Canada; bassel.akache@nrc-cnrc.gc.ca (B.A.); lise.deschatelets@nrc-cnrc.gc.ca (L.D.); blair.harrison@nrc-cnrc.gc.ca (B.A.H.); renu.dudani@nrc-cnrc.gc.ca (R.D.); felicity.stark@nrc-cnrc.gc.ca (F.C.S.); yimei.jia@nrc-cnrc.gc.ca (Y.J.); lakshmi.krishnan@nrc-cnrc.gc.ca (L.K.)

[2] Li Ka Shing Institute of Virology, Department of Medical Microbiology & Immunology, University of Alberta, 6-010 Katz Group-Rexall Centre for Health Research, Edmonton, AB T6G 2E1, Canada; landi@ualberta.ca (A.L.); llaw@ualberta.ca (J.L.M.L.); logan@ualberta.ca (M.L.); darren.hockman@ualberta.ca (D.H.); juthika@ualberta.ca (J.K.); lorne.tyrrell@ualberta.ca (D.L.T.); mhoughto@ualberta.ca (M.H.)

* Correspondence: Michael.McCluskie@nrc-cnrc.gc.ca

Received: date; Accepted: date; Published: date

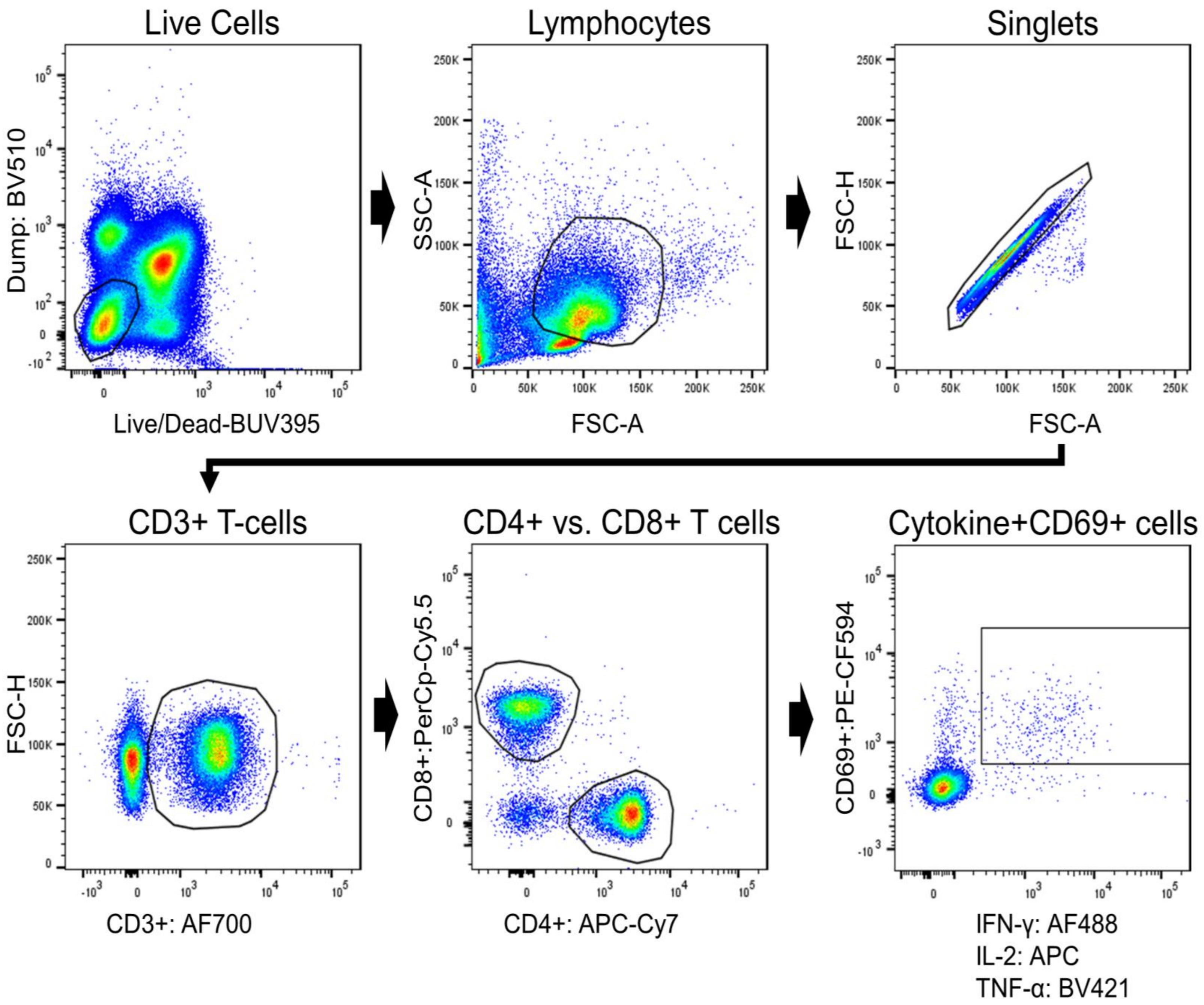
Live Cells
Dump: BV510
Live/Dead-BUV395
Lymphocytes
SSC-A
FSC-A
Singlets
FSC-H
FSC-A
CD3+ T-cells
FSC-H
CD3+: AF700
CD4+ vs. CD8+ T cells
CD8+:PerCp-Cy5.5
CD4+: APC-Cy7
Cytokine+CD69+ cells
CD69+:PE-CF594
IFN-γ: AF488
IL-2: APC
TNF-α: BV421

Figure S1. Gating strategy for intracellular cytokine staining data.

© 2019 by the authors. Submitted for possible open access publication under the terms and conditions of the Creative Commons Attribution (CC BY) license (http://creativecommons.org/licenses/by/4.0/).
